# Small Bowel Obstruction Caused by Small Intestinal Metastasis Secondary to Esophageal Carcinoma

**DOI:** 10.1155/2021/9728424

**Published:** 2021-11-18

**Authors:** Kiminari Naoshima, Keiji Abe, Kazushige Murakami, Kai Takaya, Tatsuya Nakano

**Affiliations:** ^1^Department of Surgery, Iwate Prefectural Chubu Hospital, 17-10 Murasakino, Kitakami City, Iwate 024-8507, Japan; ^2^Department of Surgery, Iwate Prefectural Tono Hospital, 14-74 Matsuzakicho, Tono City, Iwate 028-0541, Japan

## Abstract

Despite the frequent rapid spread of esophageal cancers to other organs, metastases to the small intestine are uncommon. As such, this paper describes a case of a 60-year-old male who developed a small intestinal obstruction due to metastasis from esophageal carcinoma. This patient had received radical esophagectomy for esophageal carcinoma 14 months prior to the diagnosis. Furthermore, the important role of computed tomography scans played in composing the differential diagnosis will be explored. In order to relieve the obstruction, resection of the small intestine was performed, and the patient survived six months postoperatively.

## 1. Introduction

Small bowel obstruction is a considerable cause of morbidity and mortality that often requires emergent hospital admissions. While a wide range of etiologies exist for this condition, intra-abdominal adhesions related to prior abdominal surgery account for up to 75% of cases of small bowel obstruction [1].

Less prevalent etiologies include small intestinal neoplasms, such as malignant lymphomas, primary or secondary small intestinal cancers, and local invasion by intra-abdominal malignancies. Small bowel metastasis may occur more frequently than primary small bowel malignancy [2, 3]. However, small bowel obstruction caused by secondary small intestinal tumor may be a rare entity posing a diagnostic challenge [4]. Small intestinal metastasis secondary to esophageal carcinoma is extremely rare, however should not be disregarded as a differential diagnosis when esophageal cancer patients develop small bowel obstruction.

## 2. Case Report

**Figure 1:**
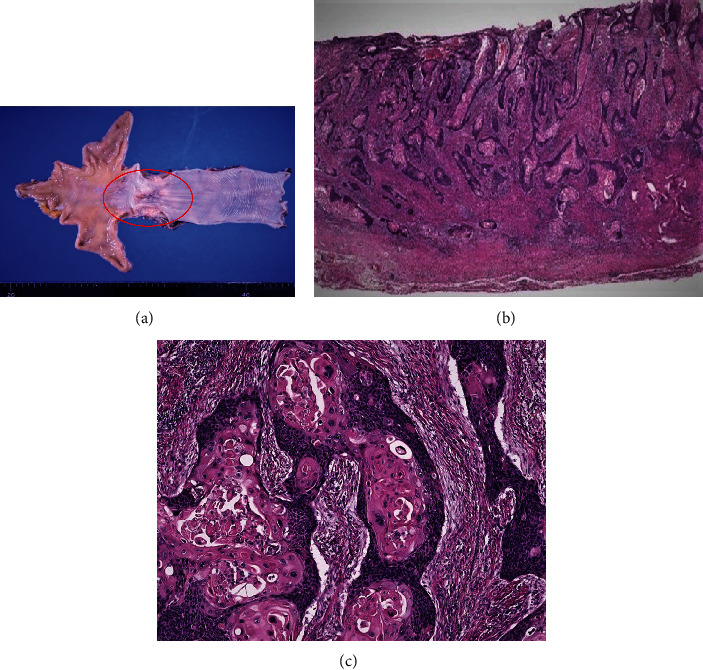
Primary esophageal carcinoma in the lower thoracic esophagus. (a) Gross appearance of the esophageal carcinoma. A 35 × 30 mm of ulcerative tumor in the lower third of the thoracic esophagus was seen. (b) Histologic finding. Hematoxylin and eosin (HE) staining showed well-differentiated squamous cell carcinoma. (c) High magnification view. Well-differentiated carcinoma with large squamous pearls was seen.

A 60-year-old man presented with a two month history of dysphagia and was referred to our hospital. He underwent upper gastrointestinal endoscopy, showing an ulcerative tumor at the lower thoracic part of the esophagus. Pathological examination of the endoscopic biopsy material revealed squamous cell carcinoma (SCC). Thus, he was diagnosed with esophageal carcinoma in the lower thoracic esophagus. All laboratory measures were within normal range. Squamous cell carcinoma (SCC) antigen and carcinoembryonic antigen (CEA) levels were 1.5 ng/dL and 3.4 mg/dL, respectively. For staging evaluation, a contrast-enhanced CT and positron emission tomography with CT were performed, and no distant metastases were detected. According to the UICC-TNM classification (version 8), the preoperative diagnosis was cStage II, SCC of the esophagus (cT3, cN0, cM0). Based on the diagnosis, neoadjuvant chemotherapy, cisplatin (80 mg/m^2^ on day 1), and a continuous infusion of 5-fluorouracil (800 mg/m^2^/day, for 5 days), were given for two cycles, followed by a radical thoracoscopic subtotal esophagectomy with two-field lymphadenectomy (abdominal and mediastinal), and an alimentary tract reconstruction was performed with an open laparotomy gastric pull-up. The gastric conduit created was raised up into the neck through the posterior mediastinal route and anastomosed with the cervical esophagus. Macroscopic analysis of the resected specimens revealed an ulcerative tumor in the lower third of the thoracic esophagus (Figure 1(a)). The pathological examination showed the primary tumor consisted of well-differentiated SCC, invading the adventitia (Figures 1(b) and 1(c)). The lymphatic invasion was evaluated as mild. The postoperative diagnosis was pStage IIB (pT3, pN0, cM0), SCC of the esophagus. Iatrogenic chylothorax occurred postoperatively on day three, necessitating ligation of the thoracic duct.

14 months after the radical esophagectomy, the patient developed acute abdominal pain and was readmitted to our hospital. Laboratory tests revealed no elevation in the serum tumor marker levels of SCC antigen (0.7 ng/dL) and CEA (1.5 mg/dL).

**Figure 2:**
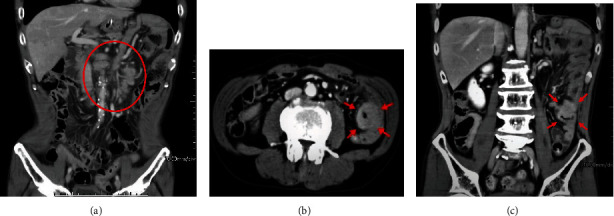
Axial and coronal images of multidetector row CT scan. (a) The preoperative CT scan showed swollen para-aortic and mesenteric lymph nodes. These were diagnosed with lymphatic metastases from the primary esophageal carcinoma. (b, c) CT scans revealed a single occlusive point. The circumferential tumor let us take into consideration an esophageal cancer metastasis as a potential cause of the obstruction. The arrows show a thickened small intestine wall, thus showing the metastatic site.

A contrast CT of the abdomen portrayed swollen para-aortic and mesenteric lymph nodes, as well as small bowel obstruction. The extent of lymphadenopathy suggested lymphatic metastases from the primary esophageal carcinoma (Figure 2(a)). Additionally, small intestinal metastasis from the esophageal carcinoma was considered to be the cause of the small bowel obstruction, as the CT scans showed a tumor in the small bowel, where circumferential mural thickening in the jejunum was visible (Figures 2(b) and 2(c)).

**Figure 3:**
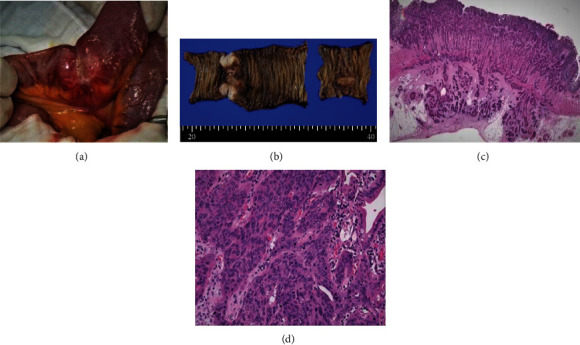
Metastatic tumors from esophageal carcinoma in the small intestine. (a) Gross finding at surgery. The metastatic tumor obstructed the small intestine. (b) Gross appearance of the resected small intestine. Two metastatic tumors were present. The left specimen showed circumferential stenosis in the jejunum. (c) Histologic finding of the metastatic tumor in the jejunum. HE stains showed moderately differentiated squamous cell carcinoma. (d) High magnification view. Moderately differentiated squamous cell carcinoma growing in a trabecular pattern. The pathological features were consistent with the diagnosis of esophageal cancer metastasis.

Surgical resection of the small intestine was performed, during which two lesions were found. One was located 40 cm distal, whilst the other 120 cm distal to the ligament of Treitz (Figure 3(b)). The proximal lesion was only a small nodule in the intestinal wall, whereas the distal tumor was larger, creating a transition point, which was considered to be the obstruction point (Figures 3(a) and 3(b)). The resected specimens were both pathologically diagnosed with moderately differentiated SCC and confirmed to originate from the esophageal carcinoma (Figures 3(c) and 3(d)).

As the patient could undergo surgery, we were able to relieve the obstruction immediately and administer docetaxel chemotherapy (70 mg/m^2^, every 21 days) with palliative intent for three months in the outpatient environment. The patient survived for six months after the small bowel obstruction surgery and died of peritoneal carcinomatosis.

## 3. Discussion

The aggressive nature of esophageal cancer often leads to its rapid spread to organs most commonly to the liver, lungs, bones, and brain [5].

Metastasis to the small intestine from esophageal cancer is rarely identified. According to the Comprehensive Registry of Esophageal Cancer in Japan, published by the Japan Esophageal Society, small intestinal metastasis occurred in less than 2% of all metastatic sites among the surgically resected esophageal cancer patients, from 1998 to 1999 [6]. Furthermore, the MEDLINE database (found through PubMed) indicated that only seven cases of small intestinal metastasis from esophageal carcinoma have been reported. As displayed in Table 1, the clinical manifestations were divided into either obstruction or perforation. Interestingly, multiple metastases were found in only two cases, including this present report. The time elapsed for small bowel metastases from the initial esophageal cancer diagnosis ranged from 0 to 24 months. Metastases were synchronous in two cases and metachronous for the other six cases. The mean time between diagnosis of the primary esophageal cancer and detection of small bowel metastases was 10 months.

While the mechanisms of metastasis from the mediastinal site to the abdominal intestinal tract are not yet completely clarified, the metastatic process is believed to involve hematogenous and lymphatic routes. In our present case, lymph node (LN) metastases were found in the mesentery of the resected intestine. Moreover, the preoperative CT scans revealed widespread LN metastases in the abdominal site, implying that the dissemination of the cancer cells took place via the lymphatic route.

Most small bowel obstructions occur as a result of prior surgeries. Intra-abdominal adhesions make up the majority of cases, followed by abdominal herniae and malignancies [1].

Primary malignant tumors of the small intestine are very rare, accounting for less than 2% of all gastrointestinal malignancies [14]. The incidence of metastases to the small bowel in autopsy cases is 2.8-8.2% [15], and they are more common than primary small bowel malignancy [2, 3]. Lobular breast cancer, lung cancer, and malignant melanoma are the most frequent primary cancers, which cause small intestinal metastasis [4, 16]. Idelevich et al. reported a literature review on small bowel obstructions caused by secondary tumors in 2006. They showed that only 36 such cases had been reported throughout a 18 year period, ranging from 1988 to 2005 [4]. The report has shown lobular breast cancer as the leading cause of metastases (17 cases), whereas lung cancer (four cases) and malignant melanoma (three cases) ranked second and third, respectively.

In this present case, above all those differential diagnoses, we strongly suspected the possibility of small intestinal metastasis from the primary esophageal carcinoma as the cause of the obstruction. The preoperative CT scans revealed a single occlusive point in the small bowel, which appeared as a circumferential thickening of the bowel wall 3 cm in length. This focal thickening of the bowel wall may be a sign of a primary or secondary tumor, arising in the bowel wall [17]. Additionally, the most commonly recurrences occur in the first two years in patients who had undergone surgical resection for esophageal carcinoma [18]. Considering the characteristic CT imaging findings and the background of the oncology patient, it was quite rational to take the small intestinal tumor, as a metastasis from the primarily treated esophageal carcinoma.

Small bowel obstruction in an oncology patient is a common but serious medical problem, associated with diagnostic and therapeutic dilemmas. Many of those patients are in advanced stages of cancer, thus necessitating a critical evaluation of surgical options to improve prognosis. In our case, the CT scan played an important role in diagnosing and determining the treatment options. It clearly showed the presence of a single occlusive point in the jejunum, which favored the operative route to resolve the bowel obstruction.

Palliative operations for relieving malignant bowel obstruction were previously reported to reduce the reobstruction rate and provide a longer symptom-free interval, compared to nonoperative management [19]. In our case, the resection of the small intestine enabled the patient a full oral intake within a few days, allowing for subsequent palliative chemotherapy relatively early, aligning with previous findings of improved quality of life in patients who underwent palliative surgery for malignant bowel obstruction [19, 20].

## 4. Conclusion

When patients with a history of esophageal carcinoma present with small bowel obstruction, it is worth considering the possibility of a small intestinal metastasis originating from the primary esophageal carcinoma. Surgical interventions may have potential to improve the prognosis of such patients.

## Figures and Tables

**Table 1:** Reported cases of small intestinal metastasis from esophageal carcinoma in international literature.

No.	Year	Author	Age	Sex	Primary esophageal carcinoma			Metastatic small intestinal tumor		Period until metastasis (months)	Treatment	Survival outcome (time after treatment)
					Location	Histology	Treatment	Symptom	Number of lesions			
1	1985	Wang [7]	65	M	Lt	SCC	Ex	Obstruction	1	18	Res	ND
2	1996	Yamada [8]	56	M	Mt	SCC	Ex→CRT	Obstruction	1	12	Res	Dead (3 years)
3	2005	Neve [9]	56	M	Lt	SCC	Ex→RT	Obstruction Perforation	1	8	Res	Alive (8 months)
4	2005	Lindenmann [10]	54	M	Mt	SCC	Ex→Chemo	Occasionally found	1	0	Res	Alive (12 months)
5	2005	Arulraj [11]	52	M	ND	SCC	CRT	Obstruction	1	5	Res	Dead (4 months)
6	2009	Dasari [12]	42	M	Lt	AC	Chemo→Ex	Obstruction	1	24	Res	ND
7	2018	Ono [13]	86	M	Mt	SCC	None	Perforation	2	0	Res	Dead (1 month)
8	2021	Present case	60	M	Lt	SCC	Chemo→Ex	Obstruction	2	14	Res	Dead (6 months)

M: male; Lt: lower thoracic esophagus; Mt: middle thoracic esophagus; SCC: squamous cell carcinoma; AC: adenocarcinoma; Ex: esophagectomy; Res: resection of the small intestine; CRT: chemoradiation therapy; RT: radiation therapy; Chemo: chemotherapy; ND: not described.

## Data Availability

The authors ensure that the datasets supporting the conclusions are included within the article.
